# CT-based radiomics and ablation margin quantification for predicting local tumor progression in Stage I NSCLC after radiofrequency ablation

**DOI:** 10.1016/j.isci.2026.117406

**Published:** 2026-09-08

**Authors:** Sibin Wang, Tianling Lyu, Zhongliang Zhang, Peng Yuan, Zenan Chen, Peng Du, Yi Zhao, Xinyuan Guo, Zichuan Jin, Yueyong Xiao, Yang Chen, Xiao Zhang

**Affiliations:** 1 Department of Radiology, The First Medical Center of Chinese PLA General Hospital, Beijing, China; 2 Chinese PLA Medical School, Beijing, China; 3 Academy for Advanced Interdisciplinary Science and Technology, Zhejiang University of Technology, Hangzhou, China; 4 Key Laboratory of New Generation Artificial Intelligence Technology and Its Interdisciplinary Applications, School of Computer Science and Engineering, Southeast University, Nanjing, China; 5 Department of Ultrasound, The Fourth Affiliated Hospital, Zhejiang University School of Medicine, Yiwu, China; 6 Department of Radiology, The Sixth Medical Center of Chinese PLA General Hospital, Beijing, China; 7 Research and Development Department, Zoye Corporation, Qingdao, China

**Keywords:** non-small cell lung cancer, computed tomography, radiofrequency ablation, local tumor progression, minimum ablation margin

## Abstract

Local tumor progression (LTP) compromises durable local control after radiofrequency ablation (RFA) for stage I non-small cell lung cancer. We developed a three-dimensional minimum ablation margin (3D MAM) quantification workflow and evaluated its integration with post-ablation CT radiomics for individualized LTP prediction. This dual-center retrospective study included 217 patients divided into training, internal validation, and external validation cohorts. Automated segmentation, deformable image registration, and Visualization Toolkit (VTK)-based analysis enabled quantitative 3D margin assessment. An insufficient MAM (<5 mm) was identified in 147 patients (67.7%). MAM ≥5 mm was independently associated with lower odds of LTP (odds ratio, 0.06; 95% confidence interval, 0.02–0.16). Among nine machine-learning classifiers, the random forest model showed consistent discrimination across cohorts, with areas under the curve of 0.877, 0.851, and 0.889. Combining quantitative margin assessment with CT radiomics may support risk-adapted surveillance after lung RFA.

## Highlights

• Three-dimensional analysis quantifies margins after radiofrequency ablation
• A minimum ablation margin of at least 5 mm predicts lower local progression
• Post-ablation computed tomography radiomics adds prognostic information
• The combined model improves prediction of local tumor progression

## Graphical abstract

**Figure:**
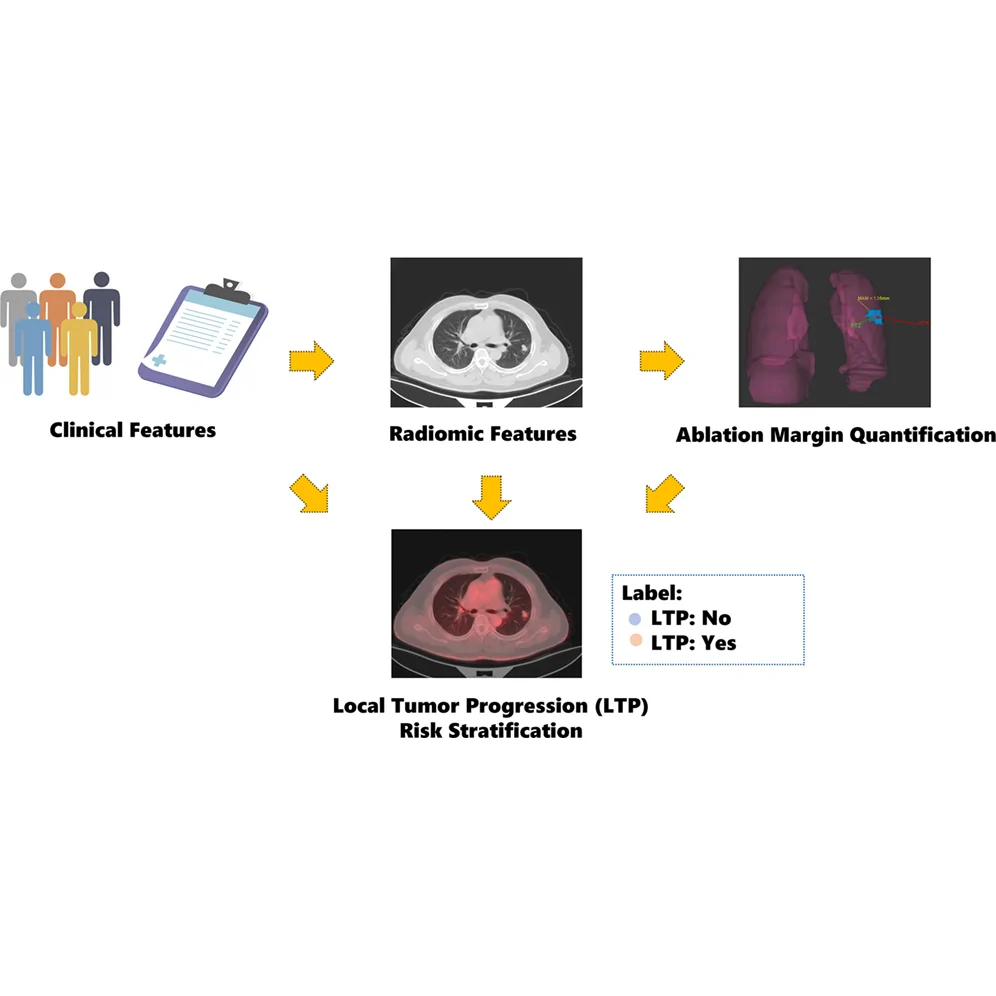

## Teaser

Oncology; Medical imaging; Artificial intelligence applications

## Introduction

CT-guided RFA is a minimally invasive local treatment for medically inoperable patients with stage I NSCLC and can preserve lung function.^1^^,^^2^ Recent guidelines and evidence syntheses support image-guided thermal ablation for patients with early-stage NSCLC, while emphasizing procedural planning, adequate ablation margins, and structured post-ablation imaging surveillance.^3^^,^^4^^,^^5^^,^^6^ Nevertheless, LTP remains an important cause of treatment failure and occurs in approximately 6%–26% of cases.^7^^,^^8^^,^^9^^,^^10^ A key determinant of local control is the minimum ablation margin (MAM), defined as the shortest distance between the treated tumor boundary and the outer boundary of the ablation zone.^11^^,^^12^^,^^13^ An insufficient margin may leave microscopic tumor extension untreated and thereby increase the risk of LTP.^14^

Current methods for assessing MAM have important limitations. Visual estimation on two-dimensional CT images is subjective, operator dependent, and prone to interobserver variability.^15^ Manual and semi-automated measurements are also time-consuming and difficult to integrate into routine clinical workflows.^16^ In addition, respiratory motion, tissue deformation, and irregular ablation geometry complicate spatial alignment between pre- and post-ablation images.^17^ These limitations reduce the reliability of margin assessment and restrict its use for postprocedural risk stratification.

Deep-learning segmentation and advanced image-processing methods may address these limitations.^18^^,^^19^ Automated segmentation can delineate the tumor and post-treatment zone, whereas deformable image registration can account for spatial displacement and tissue deformation.^17^ Together, these techniques enable three-dimensional assessment of ablation margins across serial CT examinations.^20^ Radiomic features may provide complementary information by quantifying heterogeneity within the post-treatment zone. Integrating margin measurements and radiomic features in machine-learning models may therefore improve individualized prediction of LTP.^18^

**Figure 1:**
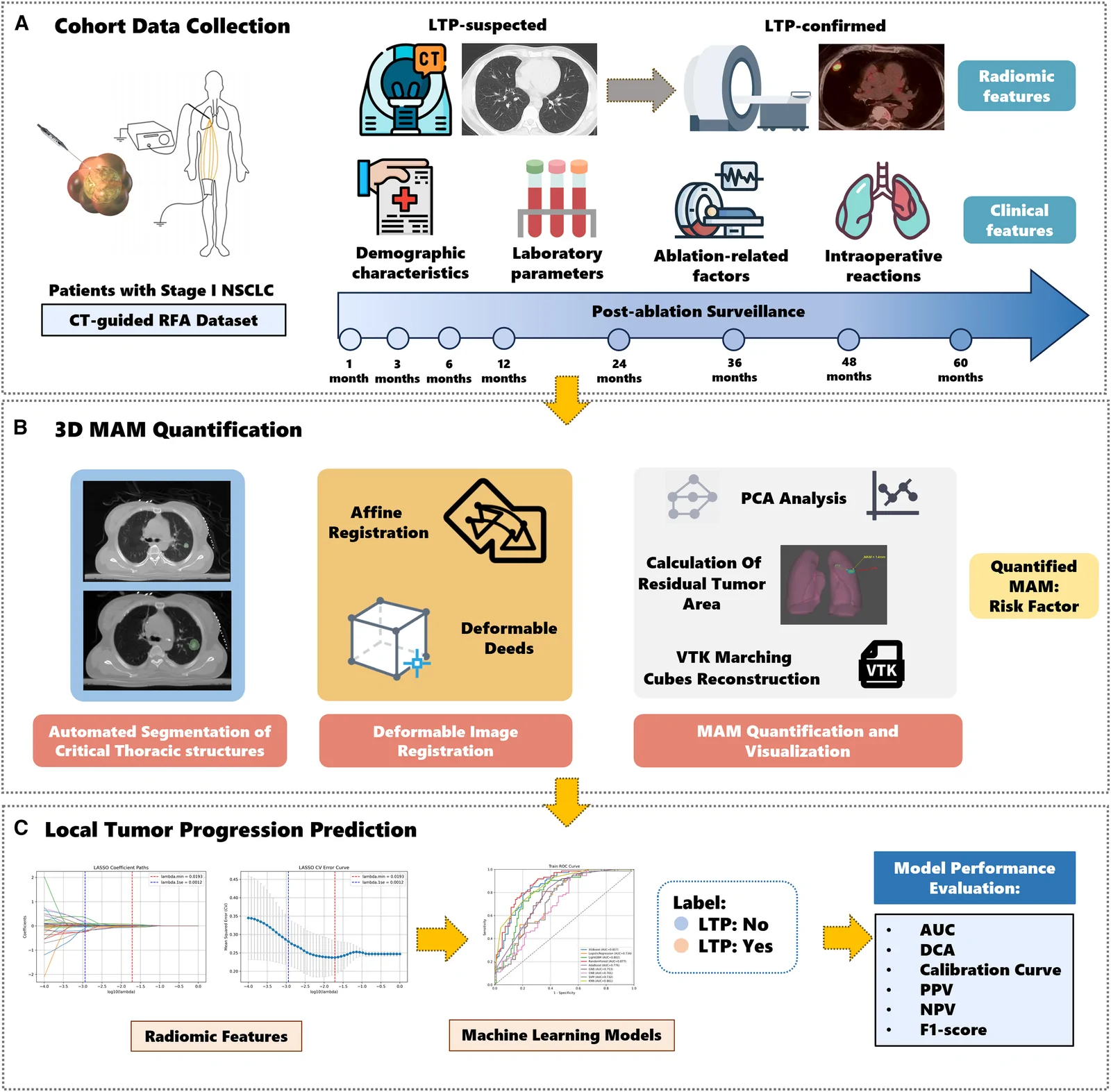
Overview of the study workflow (A) Cohort assembly. (B) Three-dimensional MAM quantification. (C) Prediction of local tumor progression.

In this dual-center study, we combined clinical data and CT imaging from patients with stage I NSCLC treated with CT-guided RFA. Automated segmentation and deformable image registration were used to align the pre-ablation gross tumor region with the immediate post-ablation post-treatment zone. We then developed a custom module based on the Visualization Toolkit (VTK) to quantify and visualize the 3D MAM.^21^^,^^22^^,^^23^ This anatomically constrained workflow enabled spatial localization of insufficient margins and prognostic stratification according to margin adequacy.^24^ To estimate individualized LTP risk, we compared nine conventional machine-learning classifiers: XGBoost, logistic regression, LightGBM, random forest, AdaBoost, Gaussian naive Bayes, complement naive Bayes, support vector machine, and k-nearest neighbors.^25^ The overall study design is shown in Figure 1.

## Results

### Patient and lesion characteristics

**Figure 2:**
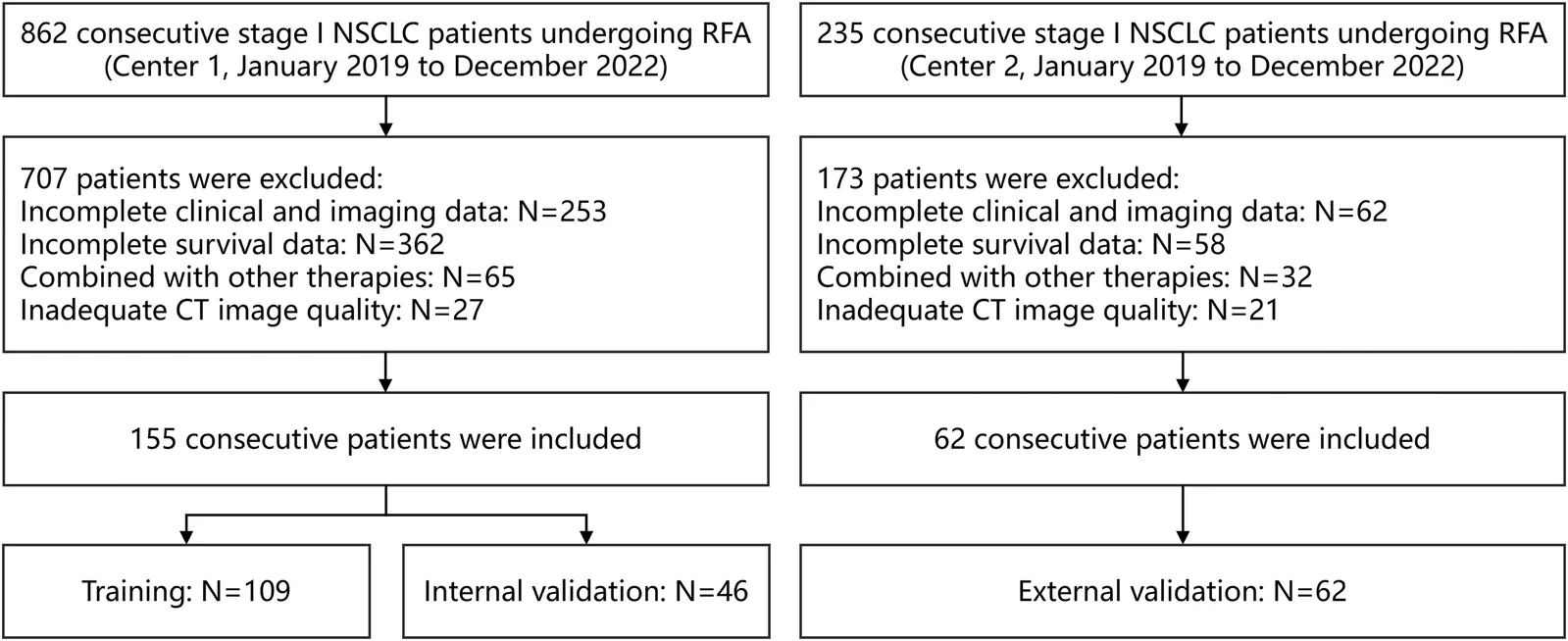
Patient selection flowchart for the cohort of patients with stage I NSCLC treated with CT-guided RFA

After application of the predefined eligibility criteria, 217 patients with stage I NSCLC who underwent CT-guided RFA were included (Figure 2). The training cohort included 109 patients from Center 1, the internal validation cohort included 46 patients from Center 1, and the external validation cohort included 62 patients from Center 2. The median follow-up was 23.0 months (interquartile range [IQR], 13.2–37.0 months). Baseline characteristics are summarized in Table 1. No statistically significant differences were observed among cohorts in age, sex, body mass index, hematologic or coagulation parameters, smoking history, comorbidities, or serum tumor markers. Adenocarcinoma was the predominant histologic subtype in all cohorts, accounting for 87.1%–91.3% of cases. Median lesion size was 18 mm (IQR, 14–23 mm) in the training cohort, 17.5 mm (IQR, 15–25.8 mm) in the internal validation cohort, and 16 mm (IQR, 13–21 mm) in the external validation cohort. Lesions were most commonly located in the upper lobes, and solid nodules were the most frequent lesion type.

**Table 1:** Baseline characteristics of the study cohorts

Characteristic	Category	Training	Internal validation	External validation	*p*_*a*_	*p*_*b*_	*p*_*c*_
Age	–	64 (56–71)	65.5 (60–69.8)	67 (61–72)	0.619	0.098	0.315
Sex	female	56 (51.4)	23 (50.0)	29 (46.8)	1.000	0.675	0.891
	male	53 (48.6)	23 (50.0)	33 (53.2)	–	–	–
BMI, kg/m^2^	–	24.0 ± 3.1	24.2 ± 3.0	24.4 ± 3.3	0.756	0.479	0.747
Smoking history	no	61 (56.0)	22 (47.8)	32 (51.6)	0.452	0.697	0.846
	yes	48 (44.0)	24 (52.2)	30 (48.4)	–	–	–
Hypertension	no	57 (52.3)	24 (52.2)	29 (46.8)	1.000	0.593	0.719
	yes	52 (47.7)	22 (47.8)	33 (53.2)	–	–	–
Diabetes	no	96 (88.1)	39 (84.8)	51 (82.3)	0.767	0.410	0.931
	yes	13 (11.9)	7 (15.2)	11 (17.7)	–	–	–
Hyperlipidemia	no	86 (78.9)	31 (67.4)	47 (75.8)	0.188	0.782	0.454
	yes	23 (21.1)	15 (32.6)	15 (24.2)	–	–	–
Histologic subtype	adenocarcinoma	99 (90.8)	42 (91.3)	54 (87.1)	1.000	0.614	0.552
	squamous cell carcinoma	10 (9.2)	4 (8.7)	8 (12.9)	–	–	–
RBC, 10^12^/L	–	4.2 (4.0–4.6)	4.3 (3.9–4.6)	4.3 (4.0–4.6)	0.896	0.353	0.617
WBC, 10^9^/L	–	6.0 (4.8–8.0)	6.1 (4.8–6.9)	6.0 (4.8–7.1)	0.750	0.547	0.754
PLT, 10^9^/L	–	205 (177–241)	217.5 (184–265.5)	202 (171–246.5)	0.107	0.916	0.138
PT, s	–	13.0 (12.5–13.4)	13.2 (12.7–13.7)	13.0 (12.5–13.6)	0.095	0.707	0.320
APTT, s	–	35.0 (32.7–37.8)	35.6 (33.3–37.6)	35.0 (31.5–36.6)	0.590	0.647	0.355
TT, s	–	16.6 (15.9–17.1)	16.6 (16.0–17.0)	16.4 (15.9–16.9)	0.840	0.235	0.419
FIB, g/L	–	3.0 (2.6–3.5)	2.9 (2.6–3.3)	3.0 (2.6–3.3)	0.697	0.826	0.773
D-dimer, mg/L	–	0.3 (0.2–0.5)	0.3 (0.2–0.4)	0.2 (0.2–0.4)	0.256	0.133	0.632
CEA	normal	89 (81.7)	40 (87.0)	53 (85.5)	0.567	0.667	1.000
	elevated	20 (18.3)	6 (13.0)	9 (14.5)	–	–	–
NSE	normal	107 (98.2)	46 (100.0)	62 (100.0)	1.000	0.535	0.124
	elevated	2 (1.8)	0 (0.0)	0 (0.0)	–	–	–
SCC	normal	104 (95.4)	45 (97.8)	59 (95.2)	0.670	1.000	0.635
	elevated	5 (4.6)	1 (2.2)	3 (4.8)	–	–	–
CYFRA 21-1	normal	92 (84.4)	37 (80.4)	50 (80.6)	0.712	0.676	1.000
	elevated	17 (15.6)	9 (19.6)	12 (19.4)	–	–	–
Lesion size, mm	–	18 (14–23)	17.5 (15–25.8)	16 (13–21)	0.240	0.266	0.053
Lesion location	RUL	39 (35.8)	17 (37.0)	24 (38.7)	0.985	0.733	0.884
	RML	10 (9.2)	3 (6.5)	3 (4.8)	–	–	–
	RLL	16 (14.7)	6 (13.0)	12 (19.4)	–	–	–
	LUL	32 (29.4)	14 (30.4)	15 (24.2)	–	–	–
	LLL	12 (11.0)	6 (13.0)	8 (12.9)	–	–	–
Lesion type	SN	47 (43.1)	19 (41.3)	25 (40.3)	0.851	0.851	0.700
	PSN	33 (30.3)	16 (34.8)	18 (29.0)	–	–	–
	PGGN	29 (26.6)	11 (23.9)	19 (30.6)	–	–	–

Continuous variables are presented as mean ± standard deviation or median (interquartile range), as appropriate; categorical variables are presented as *n* (%). *P*_*a*_ denotes the comparison between the training and internal validation cohorts; *P*_*b*_ denotes the comparison between the training and external validation cohorts; and *P*_*c*_ denotes the comparison between the internal and external validation cohorts.

BMI, body mass index; RBC, red blood cell count; WBC, white blood cell count; PLT, platelet count; PT, prothrombin time; APTT, activated partial thromboplastin time; TT, thrombin time; FIB, fibrinogen; CEA, carcinoembryonic antigen; NSE, neuron-specific enolase; SCC, squamous cell carcinoma antigen; CYFRA 21-1, cytokeratin 19 fragment; RUL, right upper lobe; RML, right middle lobe; RLL, right lower lobe; LUL, left upper lobe; LLL, left lower lobe; SN, solid nodule; PSN, part-solid nodule; PGGN, pure ground-glass nodule.

### 3D MAM quantification

**Figure 3:**
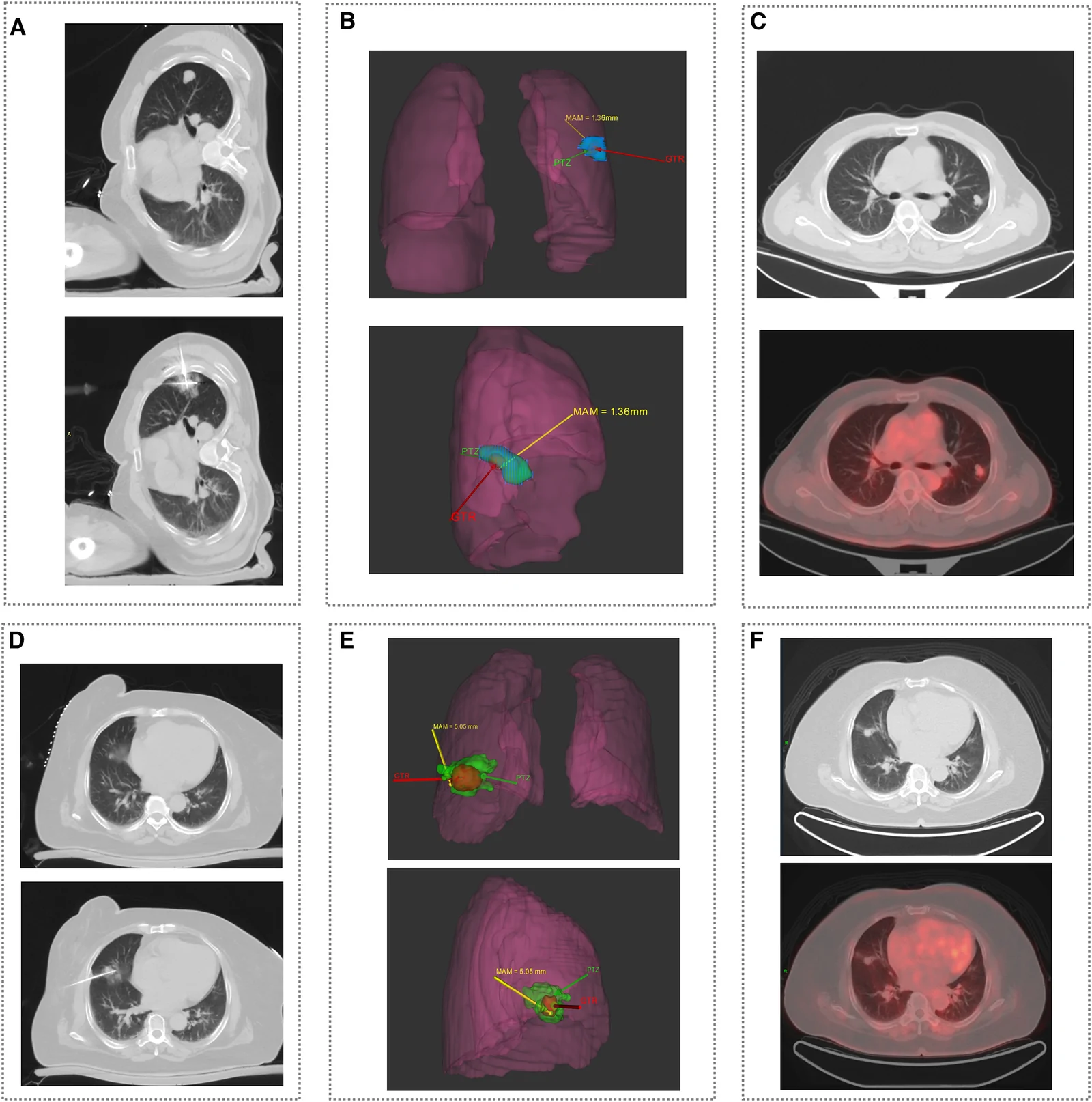
Representative three-dimensional MAM quantification in cases with insufficient and adequate margins (A) Pre-ablation and intraprocedural axial CT images from a representative case with an insufficient margin. (B) VTK-based 3D visualization of the spatial relationships among the post-treatment zone (PTZ, green contour), gross tumor region (GTR, red contour), and minimum ablation margin (MAM = 1.36 mm, yellow line). (C) Post-ablation PET/CT image shows focal residual metabolic activity in the case with an insufficient margin. (D) Pre-ablation and intraprocedural axial CT images from a representative case with an adequate margin. (E) Corresponding VTK-based 3D visualization shows complete tumor coverage with MAM ≥5 mm (representative MAM = 5.05 mm). (F) Post-ablation PET/CT image shows no focal residual metabolic activity in the case with an adequate margin.

Three-dimensional MAM quantification was completed for all 217 patients. After deformable registration of the gross tumor region (GTR) to the post-treatment zone (PTZ), the VTK-based workflow generated spatial maps of the ablation margin. Patients were classified using the prespecified 5-mm MAM threshold. An insufficient margin (<5 mm) was identified in 147 patients (67.7%). Margin deficits were most frequently located at peribronchovascular interfaces and in subpleural regions. Figure 3 shows representative cases with insufficient and adequate margins and illustrates the spatial relationships among the GTR, PTZ, and quantified MAM.

### Logistic regression analysis for LTP prediction

Variables with *p* < 0.05 in univariable logistic regression were included in the multivariable model (Table 2). In univariable analysis, MAM ≥5 mm, compared with MAM <5 mm, was associated with substantially lower odds of LTP (odds ratio [OR], 0.06; 95% confidence interval [CI], 0.02–0.15; *p* < 0.001). Lesion type also met the criterion for multivariable analysis. Compared with solid nodules (reference), pure ground-glass nodules were associated with lower odds of LTP (OR, 0.49; 95% CI, 0.25–0.96; *p* = 0.038), whereas part-solid nodules were not (OR, 0.61; 95% CI, 0.32–1.16; *p* = 0.130). No other demographic, clinical, laboratory, or imaging variable met the inclusion criterion. In the multivariable model, only MAM ≥5 mm remained independently associated with lower odds of LTP (OR, 0.06; 95% CI, 0.02–0.16; *p* < 0.001). The adjusted associations for lesion type were not statistically significant (pure ground-glass vs. solid nodules: OR, 0.85; 95% CI, 0.38–1.89; *p* = 0.694; part-solid vs. solid nodules: OR, 0.73; 95% CI, 0.36–1.49; *p* = 0.386). The adjusted OR of 0.06 corresponds to 94% lower odds of LTP in patients with MAM ≥5 mm.

**Table 2:** Univariable and multivariable logistic regression analysis for LTP prediction

Predictor	Univariable OR (95% CI)	*P*	Multivariable OR (95% CI)	*P*
**Demographic and lifestyle factors**				

Age, years	1.01 (0.99–1.04)	0.338	–	–
Male sex	1.62 (0.94–2.79)	0.083	–	–
Body mass index	1.06 (0.98–1.16)	0.161	–	–
Smoking history	1.60 (0.93–2.77)	0.089	–	–

**Comorbidities**				

Hyperlipidemia	1.26 (0.67–2.35)	0.468	–	–
Diabetes mellitus	0.89 (0.41–1.95)	0.778	–	–
Hypertension	1.17 (0.68–2.02)	0.559	–	–

**Pathology and laboratory markers**				

Squamous cell carcinoma	1.84 (0.76–4.46)	0.178	–	–
RBC	0.95 (0.53–1.69)	0.854	–	–
WBC	0.99 (0.89–1.10)	0.878	–	–
PLT	1.00 (1.00–1.01)	0.349	–	–
PT	1.16 (0.86–1.56)	0.340	–	–
APTT	1.00 (0.95–1.05)	0.989	–	–
TT	0.94 (0.77–1.13)	0.487	–	–
FIB	1.47 (0.99–2.20)	0.058	–	–
D-dimer	1.27 (0.64–2.53)	0.491	–	–
CEA elevated	1.09 (0.53–2.28)	0.809	–	–
NSE elevated	1.44 (0.09–23.38)	0.796	–	–
SCC elevated	0.71 (0.17–2.91)	0.634	–	–
CYFRA 21-1 elevated	1.20 (0.59–2.44)	0.608	–	–

**Lesion characteristics**				

Lesion size, mm	1.03 (0.99–1.07)	0.185	–	–
Lesion location (RML vs. RUL)	1.17 (0.39–3.45)	0.781	–	–
Lesion location (RLL vs. RUL)	0.93 (0.41–2.12)	0.860	–	–
Lesion location (LUL vs. RUL)	1.36 (0.69–2.66)	0.371	–	–
Lesion location (LLL vs. RUL)	0.67 (0.26–1.72)	0.401	–	–
Lesion type (PSN vs. SN)	0.61 (0.32–1.16)	0.130	0.73 (0.36–1.49)	0.386
Lesion type (PGGN vs. SN)	0.49 (0.25–0.96)	0.038	0.85 (0.38–1.89)	0.694
MAM (≥5 mm vs. <5 mm)	0.06 (0.02–0.15)	<0.001	0.06 (0.02–0.16)	<0.001

Binary predictors were coded as presence or elevated status versus the corresponding absence or normal reference category. Reference categories were adenocarcinoma for pathology, RUL for lesion location, SN for lesion type, and MAM <5 mm for the ablation-margin group. Variables with *p* < 0.05 in univariable analysis were entered into the multivariable model.

### Radiomic feature selection

**Figure 4:**
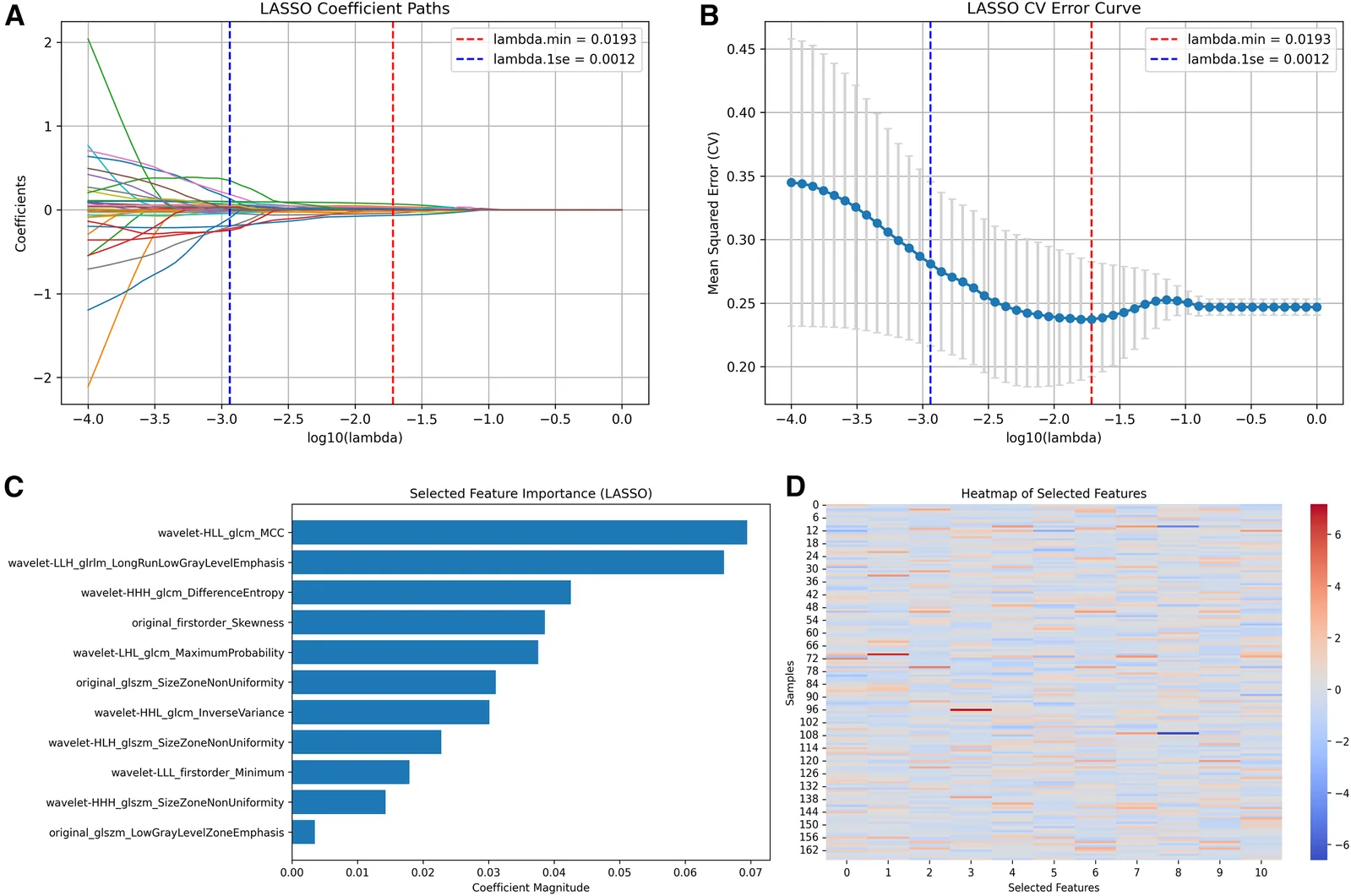
LASSO-based radiomic feature selection (A) Coefficient paths. (B) Cross-validation error curve. (C) Relative weights of the selected features. (D) Heatmap of the selected features.

After radiomic feature extraction, *Z* score normalization and Pearson’s correlation filtering were performed before 10-fold cross-validated least absolute shrinkage and selection operator (LASSO) selection. The coefficient paths and cross-validation error curve are shown in Figures 4A and 4B. At the penalty parameter that minimized the cross-validation error, LASSO retained 11 features with nonzero coefficients. The resulting signature consisted predominantly of wavelet-derived texture features from the gray-level co-occurrence matrix, gray-level run-length matrix, and gray-level size-zone matrix families, with a smaller contribution from first-order intensity features. Relative feature weights are shown in Figure 4C, and the heatmap in Figure 4D shows patient-level variation in the selected features. A radiomics score (Rad-score) was calculated as the weighted linear combination of the 11 retained features.

### Machine-learning model performance and LTP risk stratification

**Figure 5:**
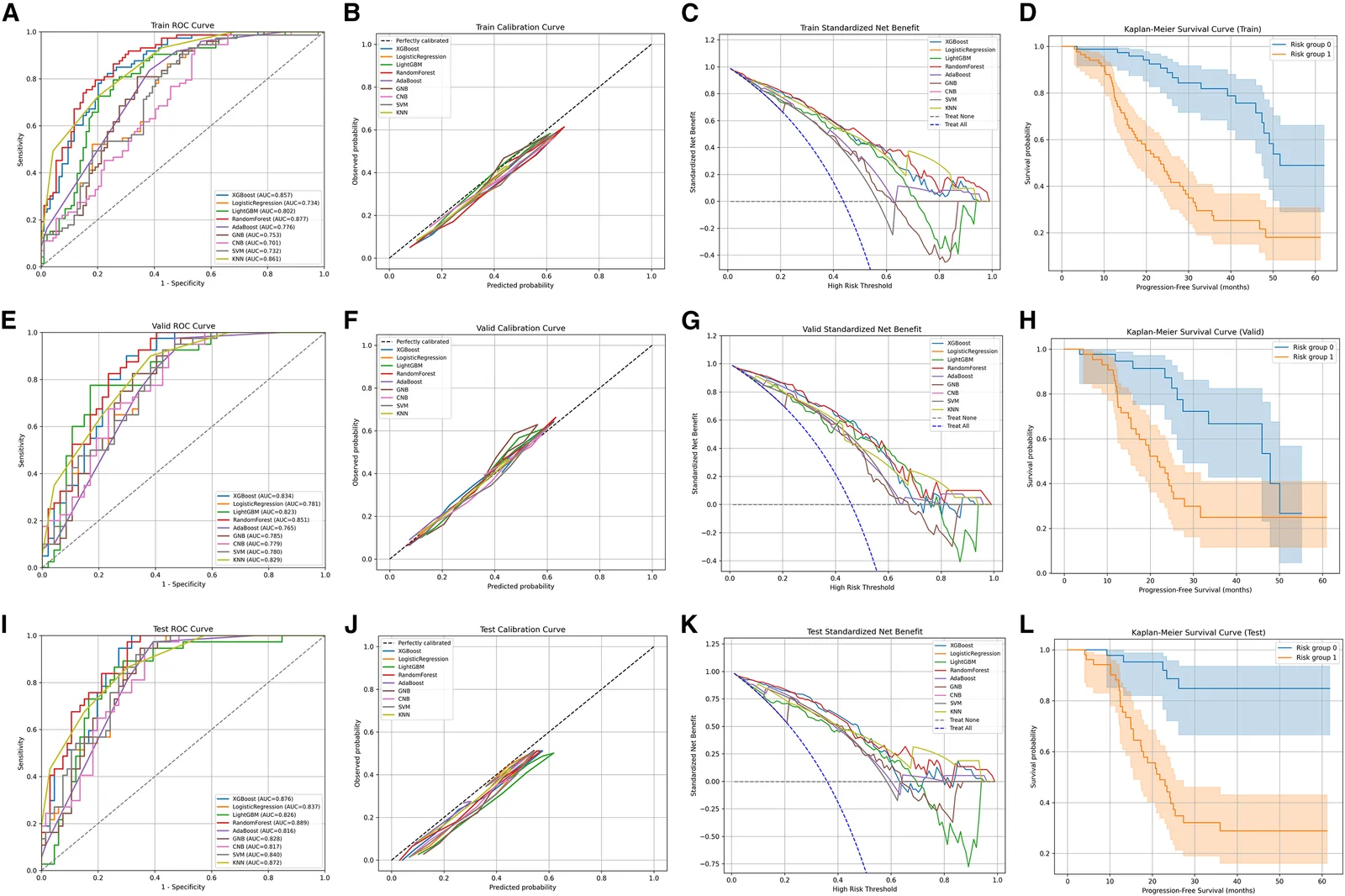
Performance of nine machine-learning classifiers across the three cohorts (A, E, and I) Receiver operating characteristic curves for the training, internal validation, and external validation cohorts, respectively. (B, F, and J) Calibration curves. (C, G, and K) Decision-curve analyses. (D, H, and L) Progression-free survival in the high- and low-risk groups defined by the optimal random forest model.

Nine machine-learning classifiers were compared across the three cohorts using measures of discrimination, calibration, and clinical utility (Figures 5A–5L and Table 3). In the training cohort, random forest achieved the highest area under the receiver operating characteristic curve (AUC, 0.877), followed by k-nearest neighbors (AUC, 0.861) and XGBoost (AUC, 0.857). LightGBM achieved an AUC of 0.802, whereas the remaining models had AUCs between 0.70 and 0.80 (Figure 5A). Random forest also had the highest AUC in the internal validation cohort (0.851), followed by XGBoost (0.834) and k-nearest neighbors (0.829) (Figure 5E). In the external validation cohort, random forest again had the highest AUC (0.889), followed by XGBoost (0.876) and k-nearest neighbors (0.872) (Figure 5I).

**Table 3:** Predictive performance of nine machine-learning models across the three cohorts

Model	Training			Internal validation			External validation		
	AUC (95% CI)	Sensitivity (95% CI)	Specificity (95% CI)	AUC (95% CI)	Sensitivity (95% CI)	Specificity (95% CI)	AUC (95% CI)	Sensitivity (95% CI)	Specificity (95% CI)
Random Forest	0.877 (0.826–0.923)	0.904 (0.831–0.969)	0.691 (0.593–0.779)	0.851 (0.762–0.924)	0.850 (0.730–0.947)	0.702 (0.564–0.830)	0.889 (0.820–0.945)	0.838 (0.714–0.952)	0.742 (0.631–0.843)
XGBoost	0.857 (0.798–0.909)	0.849 (0.763–0.929)	0.681 (0.585–0.773)	0.834 (0.748–0.911)	0.800 (0.674–0.919)	0.723 (0.595–0.846)	0.876 (0.805–0.938)	0.838 (0.700–0.946)	0.742 (0.629–0.843)
AdaBoost	0.776 (0.718–0.837)	0.918 (0.848–0.973)	0.521 (0.430–0.624)	0.765 (0.663–0.852)	0.850 (0.725–0.949)	0.596 (0.456–0.737)	0.816 (0.738–0.881)	0.892 (0.778–0.978)	0.652 (0.532–0.759)
LightGBM	0.802 (0.730–0.865)	0.726 (0.623–0.824)	0.755 (0.660–0.835)	0.823 (0.731–0.902)	0.650 (0.500–0.789)	0.851 (0.739–0.939)	0.826 (0.737–0.900)	0.811 (0.675–0.935)	0.758 (0.650–0.855)
Logistic Regression	0.734 (0.662–0.804)	0.932 (0.865–0.986)	0.447 (0.354–0.553)	0.781 (0.681–0.868)	0.925 (0.825–1.000)	0.553 (0.413–0.696)	0.837 (0.753–0.905)	0.946 (0.864–1.000)	0.621 (0.500–0.731)
KNN	0.861 (0.809–0.912)	0.726 (0.614–0.826)	0.798 (0.717–0.875)	0.829 (0.747–0.903)	0.650 (0.500–0.786)	0.787 (0.667–0.905)	0.872 (0.797–0.929)	0.676 (0.526–0.813)	0.848 (0.750–0.927)
GNB	0.753 (0.678–0.823)	0.712 (0.608–0.818)	0.681 (0.585–0.772)	0.785 (0.678–0.876)	0.675 (0.531–0.821)	0.745 (0.620–0.867)	0.828 (0.744–0.899)	0.676 (0.518–0.825)	0.758 (0.646–0.859)
SVM	0.732 (0.661–0.803)	0.932 (0.865–0.986)	0.447 (0.354–0.553)	0.780 (0.680–0.868)	0.925 (0.825–1.000)	0.553 (0.413–0.696)	0.840 (0.758–0.908)	0.946 (0.864–1.000)	0.621 (0.500–0.731)
CNB	0.701 (0.626–0.774)	0.932 (0.865–0.986)	0.447 (0.354–0.553)	0.779 (0.675–0.863)	0.925 (0.825–1.000)	0.553 (0.413–0.696)	0.817 (0.737–0.887)	0.946 (0.864–1.000)	0.621 (0.500–0.731)

AUC, area under the receiver operating characteristic curve; KNN, k-nearest neighbors; GNB, Gaussian naive Bayes; SVM, support vector machine; CNB, complement naive Bayes.

Additional classification metrics are reported in Table 4. For random forest, the positive predictive value, negative predictive value, and F1 score were 0.695, 0.903, and 0.786 in the training cohort; 0.708, 0.846, and 0.773 in the internal validation cohort; and 0.646, 0.891, and 0.729 in the external validation cohort. Calibration curves showed general agreement between predicted and observed LTP probabilities, with the random forest curves remaining close to the 45° reference line (Figures 5B–5F, and 5J). Decision-curve analysis showed a positive standardized net benefit for the better-performing models, with random forest providing the greatest net benefit across the evaluated probability thresholds (Figures 5C–5G, and 5K). Using the optimal probability threshold derived from the training cohort, Kaplan-Meier curves showed separation of progression-free survival between the low- and high-risk groups in all three cohorts (Figures 5D–5H, and 5L).

**Table 4:** Additional classification metrics of nine machine-learning models across the three cohorts

Model	Training			Internal validation			External validation		
	PPV	NPV	F1 score	PPV	NPV	F1 score	PPV	NPV	F1 score
Random Forest	0.695	0.903	0.786	0.708	0.846	0.773	0.646	0.891	0.729
XGBoost	0.674	0.853	0.752	0.711	0.810	0.753	0.646	0.891	0.729
AdaBoost	0.598	0.891	0.724	0.642	0.824	0.731	0.589	0.915	0.710
LightGBM	0.697	0.780	0.711	0.788	0.741	0.712	0.652	0.877	0.723
Logistic Regression	0.567	0.894	0.705	0.638	0.897	0.755	0.583	0.953	0.722
KNN	0.736	0.789	0.731	0.722	0.725	0.684	0.714	0.824	0.694
GNB	0.634	0.753	0.671	0.692	0.729	0.684	0.610	0.806	0.641
SVM	0.567	0.894	0.705	0.638	0.897	0.755	0.583	0.953	0.722
CNB	0.567	0.894	0.705	0.638	0.897	0.755	0.583	0.953	0.722

PPV, positive predictive value; NPV, negative predictive value; KNN, k-nearest neighbors; GNB, Gaussian naive Bayes; SVM, support vector machine; CNB, complement naive Bayes. The F1 score is the harmonic mean of precision and recall.

**Figure 6:**
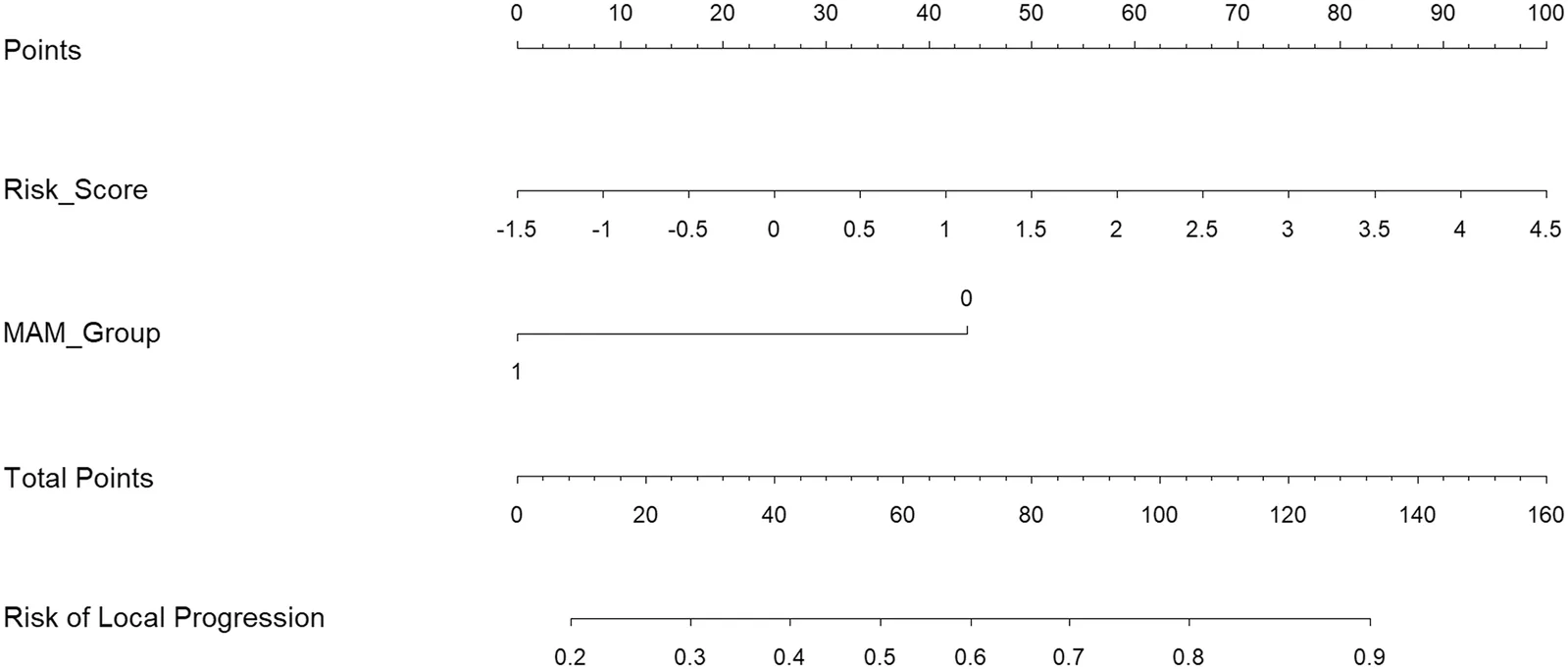
Clinical-radiomic nomogram for predicting LTP MAM_Group = 0 indicates MAM <5 mm, and MAM_Group = 1 indicates MAM ≥5 mm.

A clinical-radiomic nomogram was constructed using the Rad-score and MAM category (Figure 6). The nomogram assigns points to each predictor and converts the total score into an individualized probability of LTP. Higher total scores indicate greater predicted LTP risk.

## Discussion

This dual-center study demonstrates the feasibility and potential clinical value of 3D MAM quantification in patients with stage I NSCLC treated with CT-guided RFA. Across 217 patients and three cohorts, MAM ≥5 mm was independently associated with lower odds of LTP. Combining MAM with post-ablation radiomic features enabled individualized LTP risk stratification. Among the evaluated classifiers, random forest showed the most consistent discrimination across the training, internal validation, and external validation cohorts. The model-defined risk groups also showed clear separation of progression-free survival. These findings support the combined use of quantitative margin assessment and post-ablation imaging biomarkers for risk evaluation after lung RFA.^10^^,^^14^^,^^26^^,^^27^

The association between MAM and LTP is clinically plausible. Thermal ablation requires complete tumor coverage and an adequate circumferential safety margin to eradicate microscopic tumor extension and residual viable cells.^3^^,^^4^^,^^14^ In this study, insufficient margins were common and occurred mainly at peribronchovascular and subpleural interfaces. These locations are technically challenging during lung RFA. Adjacent vessels and airways may reduce effective thermal coverage, whereas pleural proximity can restrict electrode trajectories and energy delivery.^4^^,^^28^^,^^29^^,^^30^ Consequently, focal areas of insufficient coverage may remain even when the ablation zone appears adequate on axial CT images. Three-dimensional MAM visualization may help identify regions that require closer imaging surveillance or, when technically feasible, additional ablation.

This study also extends previous work on quantitative ablation-margin assessment. Conventional two-dimensional and manual measurements are limited by respiratory motion, tissue deformation, irregular ablation geometry, and observer dependence.^15^^,^^16^^,^^17^ The present workflow combines deformable image registration, anatomically constrained 3D margin computation, and visualization of regions with insufficient coverage. This approach is consistent with recent registration-based methods for ablation-margin quantification.^11^^,^^31^^,^^32^ Established segmentation and registration tools were used for upstream image processing, whereas the MAM quantification and visualization module was developed specifically for lung RFA. This workflow provides a more comprehensive spatial assessment of margin adequacy than slice-based visual evaluation alone.

Radiomics provided information complementary to margin quantification. Most selected features were wavelet-derived texture features, suggesting that heterogeneity within the post-treatment zone may contain prognostic information beyond lesion size and conventional CT findings. Similar observations have been reported in radiomics studies of lung ablation outcomes.^18^^,^^26^^,^^27^ This heterogeneity may reflect uneven coagulation necrosis, residual viable tissue, hemorrhage, edema, inflammation, or variable tissue response after ablation. The Rad-score summarized these imaging patterns, and its combination with MAM provided an interpretable estimate of individualized LTP risk. The performance of random forest may reflect its ability to model nonlinear associations and interactions between margin status and radiomic heterogeneity. The 5-mm cutoff was selected as a clinically interpretable benchmark and is consistent with commonly used safety-margin targets in thermal-ablation practice.^12^^,^^14^^,^^30^^,^^33^^,^^34^ This threshold can be applied to procedural planning, margin assessment, and post-ablation surveillance.

Taken together, these findings suggest two complementary clinical applications: objective identification of insufficient local coverage and individualized post-ablation risk stratification. Three-dimensional MAM quantification provides a clinically relevant measure of ablation adequacy, and MAM ≥5 mm was independently associated with lower odds of LTP. Integrating margin status with post-ablation CT radiomics may support risk-adapted surveillance after RFA in patients with stage I NSCLC.

### Limitations of the study

Several limitations should be considered. First, the retrospective dual-center design may limit generalizability. Prospective multicenter validation is required before clinical implementation. Intraprocedural CT was acquired with a 5-mm section thickness to limit radiation exposure and preserve procedural efficiency. Although all images were resampled to 1-mm isotropic voxels for computational harmonization, the original section thickness and differences between CT scanners may still affect radiomic feature stability and local margin estimates. Second, MAM quantification depends on the accuracy of segmentation, deformable image registration, and PTZ delineation. Despite expert review, small registration errors near peribronchovascular or subpleural interfaces may affect local measurements. Third, MAM was analyzed as a binary variable using a 5-mm cutoff. Future studies should evaluate MAM as a continuous predictor and assess alternative or location-specific thresholds. Finally, the cohort was restricted to stage I NSCLC treated with RFA. Further validation is needed before this workflow can be applied to metastatic lung tumors, other disease stages, or other thermal-ablation modalities.

## Resource availability

### Lead contact

Requests for further information and resources should be directed to the lead contact, Xiao Zhang (doczhang301@163.com).

### Materials availability

This study did not generate any new unique materials.

### Data and code availability

#### Data

The clinical and CT imaging data reported in this study contain sensitive human participant information and cannot be deposited in a fully public repository because of patient privacy and institutional data-governance requirements. Researchers seeking access to de-identified data should contact the lead contact. Requests will be evaluated by the data custodians and ethics committees of the participating institutions in accordance with applicable institutional policies. Access, when approved, will be limited to the data necessary for the approved research purpose and will require completion of an institutional data-use agreement.

#### Code

The original code for the VTK-based 3D MAM quantification workflow and machine-learning model evaluation is publicly available on GitHub (https://github.com/301-SibinWang/iscience).

#### Additional information

Additional information required to reanalyze the data reported in this paper is available from the lead contact upon reasonable request.

## Acknowledgments

This research was supported by the Beijing Natural Science Foundation (L252181) and the Military Commission Health Bureau Technical Product Research Project (22BJZ18 and 24BJZ16).

## Author contributions

Guarantor: X.Z. had full access to all data in the study and takes responsibility for the integrity of the data and the accuracy of the data analysis. Conceptualization: X.Z., Y.C., and Y.X.; methodology: S.W., T.L, Z.Z., and Y.Z.; software: T.L., P.Y., and Y.Z.; investigation: Z.C., P.D., X.G., and Z.J.; data curation: S.W., T.L., and Z.Z.; formal analysis: S.W.; Model development and data interpretation: S.W., T.L, Z.Z., and Y.Z.; writing – original draft: S.W. and T.L.; writing–review and editing: X.Z., Y.C., and Y.X.; supervision: X.Z., Y.C., and Y.X.; project administration: X.Z., Y.C., and Y.X.

## Declaration of interests

The authors declare no competing interests.

## Declaration of generative AI and AI-assisted technologies in the writing process

During the preparation of this manuscript, the authors used ChatGPT (OpenAI) solely to improve the language and readability. The authors reviewed and edited the output as needed and take full responsibility for the content of the manuscript.

## STAR★Methods

### Key resources table

**Table:** 

REAGENT or RESOURCE	SOURCE	IDENTIFIER
**Software and algorithms**		

TotalSegmentator	Wasserthal et al.^21^	Version 2.18.0; https://github.com/wasserth/TotalSegmentator
nnU-Net	Isensee et al.^22^	Version 2.8.1; https://github.com/MIC-DKFZ/nnUNet
DeedsBCV	Heinrich et al.^23^	Commit 00b4653; https://github.com/mattiaspaul/deedsBCV
Visualization Toolkit (VTK)	Kitware	Version 9.7.0; https://gitlab.kitware.com/vtk/vtk
PyRadiomics	PyRadiomics	Version 3.1.0; https://github.com/AIM-Harvard/pyradiomics
R	R Foundation	Version 4.6.1; https://www.r-project.org/
Python	Python Software Foundation	Version 3.11; https://www.python.org/
Custom VTK-based 3D MAM quantification and machine-learning evaluation code	This paper	https://github.com/301-SibinWang/iscience

### Experimental model and study participant details

#### Study design and setting

This retrospective dual-center cohort study was conducted at the First Medical Center and the Sixth Medical Center of the Chinese PLA General Hospital. Consecutive patients with stage I NSCLC who underwent CT-guided RFA between January 2019 and December 2022 were screened for eligibility. Patients from the First Medical Center were randomly divided into training and internal validation cohorts in a 7:3 ratio using a computer-generated sequence. Patients from the Sixth Medical Center formed the external validation cohort. All included patients were Han Chinese. Tumors were staged according to the International Association for the Study of Lung Cancer staging system. Patient clinical and demographic characteristics are presented in Table 1.

The study protocol was approved by the ethics committees of both participating centers: the First Medical Center (approval no. S2023-695-01) and the Sixth Medical Center (approval no. HZKY-PJ-2023-37). Both committees waived the requirement for informed consent because of the retrospective study design.

#### Inclusion and exclusion criteria

Patients were eligible if they met all of the following criteria: (1) histologically confirmed stage I NSCLC according to the 9th edition of the International Association for the Study of Lung Cancer staging system; (2) CT-guided RFA performed with curative intent; (3) available preprocedural, intraprocedural, and postprocedural CT images of sufficient quality for segmentation and registration; and (4) complete baseline clinical and follow-up data. Exclusion criteria were (1) previous treatment for NSCLC, including surgical resection or radiotherapy; (2) active severe infection or clinically significant coagulopathy; and (3) inadequate CT image quality or postprocedural changes that precluded reliable segmentation or registration.

### Method details

#### Data collection and outcome assessment

Baseline clinical data were retrospectively extracted from electronic medical records, and imaging data were retrieved from the institutional picture archiving and communication systems. Study variables included demographic characteristics, laboratory measurements, and imaging features. LTP was assessed according to modified Response Evaluation Criteria in Solid Tumors (*m*-RECIST) and was defined as a ≥20% increase in the sum of the longest diameters of the target lesion or the appearance of a new lesion.^26^^,^^35^^,^^36^

The endpoint was assessed independently by two radiologists with experience in thoracic imaging and lung ablation. Disagreements were resolved by consensus, with adjudication by a senior radiologist when required. Progression-free survival (PFS) was defined as the interval from RFA to radiologically confirmed LTP.^26^^,^^35^^,^^36^ Surveillance was performed primarily with contrast-enhanced chest CT.^3^^,^^4^ The first follow-up examination was performed 1 month after ablation, followed by scheduled examinations at 3, 6, and 12 months. Patients without LTP during the first year underwent annual CT surveillance thereafter. PET/CT was used to confirm suspected LTP detected during routine follow-up.

#### RFA procedures and image acquisition

All RFA procedures were performed under CT guidance using the Cool-tip RFA system (Medtronic, USA). Imaging was performed with Philips Brilliance CT or GE Discovery CT scanners. Standardized acquisition parameters were used on both platforms: tube voltage, 120 kV; tube current–time product, 250 mAs; section thickness, 5 mm; and section interval, 5 mm for intraprocedural and immediate postprocedural imaging. For segmentation, registration, MAM quantification, and radiomics analysis, all images were resampled to 1-mm isotropic voxels. The same preprocessing pipeline was applied to the training, internal validation, and external validation cohorts. Ablation power (20–40 W) and duration (12–21 min) were adjusted during the procedure according to real-time impedance, tumor size, morphology, and location.^36^

#### Automated segmentation of thoracic structures

The gross tumor region (GTR), post-treatment zone (PTZ), and relevant thoracic structures were segmented on pre- and post-ablation CT images using TotalSegmentator, a deep-learning segmentation tool based on the nnU-Net architecture.^21^^,^^22^ All automated segmentations were reviewed and manually corrected when necessary before downstream analysis. The GTR was defined on the pre-ablation planning CT and served as the tumor-side reference for MAM calculation. The expert-reviewed PTZ on the immediate post-ablation CT served as the treatment-side boundary.

#### Deformable image registration

Deformable image registration was performed with the DeedsBCV implementation. Each pre-ablation CT volume containing the GTR was registered to the corresponding immediate post-ablation CT volume containing the PTZ. DeedsBCV was selected because it accommodates local displacement, sliding motion at organ interfaces, and intensity variation associated with tissue deformation. The algorithm uses discrete optimization within a Markov random field framework.^23^ Experienced radiologists assessed the resulting deformation fields and spatial alignment by reviewing fused pre- and post-ablation images for anatomic plausibility.

#### MAM quantification and visualization

MAM was quantified using a custom module developed with VTK. For prognostic stratification, MAM was dichotomized at 5 mm in accordance with commonly used thermal-ablation margin targets.^12^^,^^30^^,^^33^^,^^34^ After deformable registration, the registered pre-ablation GTR served as the geometric tumor reference. The visible tumor boundary was not redelineated on the immediate post-ablation CT.^11^^,^^12^^,^^31^^,^^32^ Cases with inadequate image quality for reliable PTZ delineation or anatomically plausible registration were excluded according to the predefined image-quality criterion. Binary masks of the GTR and PTZ were converted into high-resolution surface meshes using the VTK Marching Cubes algorithm. To restrict margin assessment to the lung parenchyma, an anatomically constrained 3D region-growing algorithm was initialized from the GTR surface and guided by the lung mask.^37^ The lobar mask further restricted region growing to the visceral pleural boundary. Regions of potential insufficient coverage were identified using 3D Boolean subtraction of the PTZ from the registered GTR. Connected-component analysis was then used to separate these regions for geometric characterization. For each residual component, principal component analysis was used to estimate its dominant orientation and define two parallel tangent planes. The minimum orthogonal distance between the planes was calculated as the smallest dimension of an oriented bounding box fitted to the component. This value represented the thinnest residual component dimension.

#### Radiomics and machine-learning model development

For radiomics analysis, the region of interest was the expert-reviewed PTZ on the immediate post-ablation CT. Before feature extraction, all images were resampled to 1-mm isotropic voxels. Radiomic features were extracted using PyRadiomics and standardized using z scores. Highly correlated features were removed by Pearson correlation filtering before penalized feature selection. LASSO was then applied, and the optimal regularization parameter was selected by 10-fold cross-validation using the minimum mean squared error criterion. For each patient, the Rad-score was calculated as the weighted sum of the selected features, with the LASSO coefficients used as weights. Nine conventional machine-learning algorithms were trained and compared: XGBoost, logistic regression, LightGBM, random forest, AdaBoost, Gaussian naive Bayes, complement naive Bayes, support vector machine, and k-nearest neighbors. Feature selection and model development were performed in the training cohort. The internal and external validation cohorts were held out for model evaluation. A nomogram based on the selected predictors was generated to estimate individualized LTP probability.

### Quantification and statistical analysis

Continuous variables are reported as mean ± standard deviation or median (interquartile range), as appropriate. Categorical variables are reported as frequencies and percentages. Baseline characteristics were compared among cohorts using one-way analysis of variance, the Kruskal–Wallis test, the *χ*^2^ test, or Fisher’s exact test, as appropriate. In the training cohort, potential clinical predictors of LTP were evaluated using univariable and multivariable logistic regression. Variables with *p* < 0.05 in univariable analysis were entered into the multivariable model. All tests were two-sided, and *p* < 0.05 was considered statistically significant. Radiomic features were standardized using z scores. Pearson correlation filtering and 10-fold cross-validated LASSO were used for dimensionality reduction and Rad-score construction. Model discrimination was evaluated using the AUC, sensitivity, specificity, positive predictive value, negative predictive value, and F1 score. Calibration curves were used to compare predicted and observed LTP probabilities. Decision-curve analysis was used to evaluate standardized net benefit. The optimal probability threshold derived from the training cohort was used to classify patients as low or high risk. PFS was compared using Kaplan–Meier analysis. Conventional statistical analyses were performed in R (version 4.6.1), and machine-learning analyses were implemented using standard open-source Python packages.
